# Forms and correlates of child maltreatment among autistic children involved in child protection services

**DOI:** 10.3389/frcha.2024.1386781

**Published:** 2024-08-21

**Authors:** Jacinthe Dion, Geneviève Paquette, Mireille De La Sablonnière-Griffin, Malena Argumedes, Alexa Martin-Storey, Marie-Louise Bolduc, Sonia Hélie, Ève-Line Bussières

**Affiliations:** ^1^ Department of Psychology, Université du Québec à Trois-Rivières, Trois-Rivières, QC, Canada; ^2^ Interdisciplinary Research Center on Intimate Relationship Problems and Sexual Abuse (CRIPCAS), Montreal, QC, Canada; ^3^ Department of Psychoeducation, Université de Sherbrooke, Sherbrooke, QC, Canada; ^4^ Unité Mixte de Recherche INRS-UQAT en études Autochtones, Institut National de la Recherche Scientifique, Val D’Or, QC, Canada; ^5^ CIUSSS du Centre-Sud-de-L’Île-de-Montréal, Institut Universitaire Jeunes en Difficulté, Montreal, QC, Canada

**Keywords:** child abuse, neglect, autism, disability, child welfare, intellectual disability

## Abstract

**Background:**

Child maltreatment is a significant social problem impacting both health and society, with severe and enduring consequences. Certain children, such as those with neurodevelopmental conditions like autism, may be more at risk of experiencing maltreatment. However, little research has examined the characteristics of these children. This study aimed to compare child maltreatment and child protection services experienced by autistic children to those of non-autistic children.

**Method:**

Drawing from a representative selection of verified cases of child abuse investigated by child protection services in Quebec, Canada, a sample of 1,805 substantiated child maltreatment cases were analyzed.

**Results:**

Overall, 4.0% (*n* = 73) of children had child protection services-reported autism diagnoses. Attention-deficit (OR = 2.207) and attachment problems risk (OR = 2.899) were higher among autistic children compared to non-autistic children. They were more likely to be boys (OR = 5.747), and to present with an intellectual disability (OR = 11.987), but less likely to have previously been investigated by child protection services (OR = 0.722).

**Conclusion:**

These findings suggest that autistic children who have been maltreated are facing specific challenges that require protective interventions tailored to their specific needs.

## 1 Introduction

Child maltreatment is a significant and complex issue impacting both health and society, with severe and enduring consequences (1). As per the World Health Organization (WHO), “Child maltreatment encompasses various forms of physical and emotional mistreatment, sexual exploitation, and neglect, leading to actual or potential harm to a child's well-being, growth, or dignity within the framework of a relationship characterized by responsibility, trust, or authority” (2). Certain children are more likely to experience child maltreatment. In particular, studies have found that children with neurodevelopmental conditions, such as autism, are more likely to be maltreated or sexually abused than their non-autistic peers (3, 4). While the global prevalence of autism is about 1% (5), some studies indicate that the proportion of autistic children is twice as high among children who have experienced maltreatment (6, 7). In addition, research indicates a higher proportion of reported and substantiated maltreatment among autistic children compared to non-autistic children (8, 9). Little research, however, has identified the individual or systemic risk factors for maltreatment among autistic children. And, while establishing risk factors related to child maltreatment among autistic children is important, intervention efforts require information about use of protective services among these children. The aim of this study, therefore, was to fill these knowledge gaps by investigating the characteristics of child maltreatment and of child protection services (CPS) experienced by children with a CPS-reported autism diagnosis.

The ecological model, when applied to the abuse of children with disabilities (10), provides a framework for better understanding child maltreatment for these populations. According to this framework, greater vulnerability for maltreatment results from interactions between the child, their parents, their family characteristics, and broader structural factors. Increasing understanding of maltreatment among autistic children requires an approach that encompasses the factors traditionally studied in both the child maltreatment and the child disability fields (10). For example, autistic children often present with co-occurring mental health conditions (e.g., repetitive patterns of behavior, self-destructive behaviors, anxiety, hyperactivity) (11). Gillberg (12) also argues that autistic children often present with overlapping dysfunctions/neurodevelopmental problems, which increase their needs and therefore, require a holistic approach to intervention. Their specific needs may in return increase the level of stress experienced by parents, stress levels which could be further exacerbated in low socioeconomic settings or when lower levels of social support is available to the family (13, 14). The needs of autistic children may be difficult to address in families where there are parent and family-related problems (e.g., mental health issues, disrupted parent-child attachment), increasing the risk of maltreatment. Highlighting their specific vulnerability, autistic children involved in CPS are more likely to present with mental health conditions compared to children presenting to CPS with other neurodevelopmental conditions or without conditions (7).

Research provides some empirical support to the ecological model as a framework for understanding maltreatment among autistic children. Autistic children, like their non-autistic peers, experience a wide range of mental health conditions such as post-traumatic stress symptoms (15), and internalizing and externalizing behaviors problems (9, 16, 17). Research also suggests that maltreated autistic children often present with higher levels of psychological difficulties than non-autistic maltreated children (9, 15–17). In a representative cross-sectional sample of 8,192 nine-year-old twins born in Sweden, maltreated children exhibited a higher prevalence of neurodevelopmental disorders, including autism, compared to non-maltreated children (8). They were also at increased risk of presenting with multiple neurodevelopmental disorders, adding to this elevated risk comorbidity. Nonetheless, it may be difficult to disentangle the correlates or consequences of abuse from difficulties experienced by autistic children, because autism often co-occurs with mental health and behavioral conditions such as anxiety, attention deficit/hyperactivity disorder (ADHD/ADD), depression (18–20). Results of a systematic review of 47 studies involving adults with autism also revealed a high prevalence of several psychiatric conditions, with the most common being ADHD, depression, and anxiety disorders (21). These researchers also conducted a meta-analysis with the 18 studies reporting prevalence for any psychiatric disorders and found that 54,8% of autistic adults had another psychiatric disorder. Moreover, children's post-abuse adaptation may be influenced by the family's reactions to the abuse (e.g., belief or denial, support) and the social context (e.g., poverty, isolation, invisibility of disabled children in mainstream services, lack of voice and agency) (22). Some sequelae may also be undiagnosed since some autistic children may have a reduced capacity to express emotions and talk about their psychological state (23). In addition, results of the small body of literature conducted to better understand the experiences of autistic children in CPS indicate that autistic children are more likely to receive services from the child protection system (7) and to experience out-of-home placement (24).

Overall, the limited research documenting the characteristics of maltreated autistic children indicates that they differ from other maltreated children. However, these studies are not without shortcomings, limiting their generalizability.^1^ For instance, some studies did not distinguish between the characteristics of autistic and non-autistic children according to the forms of child maltreatment (reported/investigated/substantiated), i.e., all maltreatment situations were considered as a monolith group which limits our capacity to understand if autistic children experience specific forms of maltreatment at higher, similar or lower rates than non-autistic children (6, 8, 25, 26). Others examined only individual-level factors, such as children's mental health symptoms, or aspects of the maltreatment (e.g., duration, number of perpetrators) (6, 9, 15–17, 25, 26). Additional limitations include the use of convenience samples and studies conducted using only bivariate analysis (7, 16). Moreover, only a few studies have compared populations of autistic children to those without autism among confirmed victims of maltreatment. It is crucial, then, to increase our knowledge of how autistic children differ from non-autistic children, as they may require more tailored support from child protection workers, either because of their intellectual functioning, communication limitations (23), co-occurring psychiatric and behavioral disorders (7) or because of differences in their socioenvironmental context (27).

Drawing from a representative selection of verified cases of child abuse investigated by CPS in a specific Canadian province, this research endeavor seeks to contribute to additional knowledge to this growing research area by enhancing our comprehension of the characteristics of the abuse incidents, as well as family and child-level characteristics of children investigated by CPS according to the presence of a CPS-reported autism diagnosis. To be more precise, focusing on children who have experienced abuse or neglect and were identified by CPS, this cross-sectional study seeks to compare cases with autistic children to those with non-autistic children focusing on socio-demographic and functional aspects, as well as the attributes of their caregivers, the forms of confirmed child maltreatment experienced, and a selection of targeted child protective service factors.

## 2 Materials and methods

### 2.1 Study context

This research involved the secondary utilization of data originating from the 2014 Quebec Incidence Study (QIS-2014 (28); regarding cases investigated by Child Protective Services (CPS). Out of all the child reports (aged 0–17) examined between October 1 and December 31, 2014, a random 50% were chosen to participate in the QIS-2014, resulting in a final study cohort of 4,011 children. This random selection criterion of 50% was employed to ensure the sample's representativeness. Approval was obtained through an ethics certificate from the institutional review boards of the Centre jeunesse de Montréal-Institut universitaire and the Centre jeunesse de Québec-Institut universitaire. A comprehensive description of the incidence study is available in Hélie et al. (28).

### 2.2 Brief review of CPS decision-making and intervention process in QIS-2014

Incidents of suspected child maltreatment are initially reported to CPS, where they undergo a preliminary assessment to determine whether a full investigation is warranted. Reports identified for further examination are probed to address two primary inquiries: whether the allegations are confirmed, and if so, whether the child's safety and development are in jeopardy. In cases where the child's safety and development are at risk, protective measures, either court-mandated or voluntary, are put into effect. These measures can involve home-based CPS services or placing the child outside the home. In contrast, when a report is not flagged (for example, when the investigation concludes that the situation is unsubstantiated or when the case is confirmed but the child's safety and development remain unimpaired), the child and their family may be directed to appropriate public and community resources as necessary.

### 2.3 Sample

Three selection criteria were employed to establish the sample for the present study: (1) as Autism Spectrum Disorder is less frequently diagnosed within the first two years of life (29), only children aged 2–17 years were considered; (2) child maltreatment had to be substantiated for at least one of the following four forms: physical abuse, sexual abuse, neglect, or psychological maltreatment (including exposure to intimate partner violence); and (3) data regarding autism diagnose as reported by CPS workers had to be collected (20 children were excluded due to the caseworker noting the presence of autism as “unknown”). The final study sample comprised 1,805 children (Mage = 9.2 years, SD = 4.1; 48.0% girls). Most of children's mother spoke French (85.7%). The majority of children had white primary caregiver (76.3%). The other ethno-cultural groups identified were: Indigenous (2.7%), Asian (7.2%), African American/Black (10.2%), Hispanic/Latino (2.9%), and other/missing (0.7%). Just over a half of the children lived in a family where the source of income was from working full time by at least one of the primary caregivers (53.0%).

### 2.4 Data collection, measurement instrument, and variables collected

Caseworkers who were assigned to investigate the chosen reports were mandated, based on their current knowledge of the cases, a validated 50-item questionnaire developed for research purpose via a secure online platform (28). The questionnaire encompassed inquiries about the investigated instances of alleged maltreatment, the attributes of the child, caregivers, and the household, as well as CPS services. Caseworkers had the option to indicate the mental health and/or behavioral conditions of the child or one of the caregivers when there was a professional diagnosis, a self-reported claim by the child or caregiver, or when there were substantial grounds for inclusion in the written report or discussion of the case. More specifically, caseworkers were asked to report if the functioning problems identified were “confirmed,” i.e., diagnosed by a professional or directly observed, or “suspected,” i.e., the suspicions were sufficient to record it as part of their assessment. The categories for confirmed or highly suspected conditions for all key clinical variables (as well as for autism) were collated for the analyses, as done previously (30). Because child welfare workers documented only functioning issues that they became aware of during their investigation, the occurrence of autism was likely underestimated. Caseworkers participating in the study (approximately 700 in total) received a half-day training session along with accompanying guidelines, and an accompanying codebook defined all items collected. Variables used in this current study are all presented in Table 1.

**Table 1:** Results of the descriptive analyses and the univariate analyses comparing maltreated autistic children to children without autism.

Variables	Descriptive analyses		Bivariate analyses	
	Without autism (*n* = 1,732)	With autism (*n* = 73)	B (SE)	OR [95% CI]
Individual				
Boys	50.7%	83.6%	1.598 (0.32)***	4.944 [2.644–9.247]
Age	*x* = 9.23 (SD = 4.2)	*x* = 8.53 (SD = 4.0)	−0.041 (0.03)^t^	0.960 [0.907–1.017]
Physical abuse substantiated	30.4%	28.8%	−0.080 (0.26)	0.923 [0.551–1.549]
Sexual abuse substantiated	5.1%	2.7%	−0.642 (0.73)	0.526 [0.127–2.181]
Neglect substantiated	37.7%	47.9%	0.420 (0.24)^t^	1.522 [0.952–2.433]
Psychological maltreatment substantiated	41.9%	30.1%	−0.515 (0.26)*	0.598 [0.359–0.994]
Multiple maltreatment events	75.8%	76.7%	0.051 (0.28)	1.052 [0.605–1.830]
Depression or anxiety in the child	30.4%	40.8%	0.458 (0.25)^t^	1.581 [0.974–2.566]
Suicidal thoughts by the child	8.3%	8.3%	0.006 (0.44)	1.006 [0.429–2.360]
Self-destructive behaviors of the child	8.6%	16.7%	0.757 (0.33)*	2.132 [1.122–4.053]
ADD/ADHD in the child	32.2%	66.7%	1.436 (0.26)***	4.203 [2.548–6.933]
Attachment disorder in the child	16.4%	41.7%	1.289 (0.25)***	3.630 [2.233–5.901]
Aggressive behaviors of the child	11.9%	24.7%	0.886 (0.28)**	2.425 [1.397–4.211]
Repeated runaways by the child	3.7%	1.4%	−1.019 (1.02)	0.361 [0.049–2.640]
Inappropriate sexual behaviors of the child	5.2%	4.2%	−0.225 (0.60)	0.798 [0.246–2.586]
Physical disability in the child	1.6%	5.5%	1.296 (0.55)*	3.654 [1.244–10.732]
ID in the child	3.6%	30.6%	2.453 (0.29)***	11.622 [6.632–20.366]
Environmental				
Family composition: 2 parents	43.4%	42.5%	−0.039 (0.24)	0.962 [0.599–1.545]
Source of family income other than work	40.0%	46.6%	0.268 (0.24)	1.307 [0.817–2.091]
Use of corporal punishment on the child	29.2%	29.4%	0.012 (0.27)	1.012 [0.594–1.724]
Alcohol abuse by one of the caregivers	13.9%	12.3%	−0.134 (0.36)	0.874 [0.429–1.780]
Drug abuse by one of the caregivers	16.9%	13.7%	−0.249 (0.35)	0.780 [0.395–1.537]
ID in one of the caregivers	2.9%	5.5%	0.668 (0.53)^t^	1.950 [0.685–5.553]
Mental health problem in one of the caregivers	28.1%	42.5%	0.638 (0.24)**	1.892 [1.176–3.045]
Physical health problem in one of the caregivers	10.5%	19.2%	0.704 (0.31)*	2.021 [1.106–3.692]
Lack of social support for one of the caregivers	36.8%	56.2%	0.790 (0.24)**	2.202 [1.373–3.533]
Intimate partner violence in one of the caregivers	27.5%	34.2%	0.315 (0.25)^t^	1.370 [0.835–2.248]
Report from a professional	80.1%	79.5%	−0.043 (0.30)	0.958 [0.537–1.711]
Alleged perpetrator of the maltreatment is a caregiver	80.1%	82.2%	0.134 (0.31)	1.144 [0.621–2.108]
Service-related				
Decision: safety or development compromised	47.9%	60.3%	0.502 (0.24)*	1.653 [1.025–2.666]
Number of past investigations	*x* = 0.95 (SD = 1.42)	*x* = 0.70 (SD = 0.98)	−0.153 (0.10)^t^	0.858 [0.701–1.051]
Referral to another service	58.7%	64.4%	0.240 (0.25)	1.271 [0.780–2.071]
Police involvement	33.1%	32.9%	−0.008 (0.25)	0.992 [0.602–1.632]
Placement during investigation	14.9%	13.7%	−0.095 (0.35)	0.910 [0.461–1.796]

B, coefficient *b*; SE, standard error; OR, odd ratio; CI, confidence interval; x, mean; SD, standard deviation.

^t^ *p* < .25; *p < .05; **p < .01; ***p < .001.

#### 2.4.1 Autism

The autism variable was employed as the dependent variable for comparing the children within the sample. In the current study, a child was classified as having autism if the question “the child received a diagnosis for autism spectrum disorder/pervasive developmental disorder” was answered affirmatively.

#### 2.4.2 Child under investigation

Variables in this category included age, whether the child was a boy or a girl, form of maltreatment experienced (physical abuse, sexual abuse, neglect, and psychological maltreatment; a child can be indicated to have experienced up to three distinct forms of maltreatment during a single investigation), whether maltreatment consisted of repeated events, and child's mental health conditions [depression, suicidal thoughts, self-destructive behaviors, ADD/ADHD, attachment disorder, aggressive behaviors, repeated running away, inappropriate sexual behaviors, physical disabilities, and intellectual disabilities (ID)]. Given that none of the autistic children were reported to experience alcohol abuse or drug abuse, these variables were not included in the analyses. In addition, because of diagnostic overlap, learning disabilities and developmental delays were also dropped from the analyses.

#### 2.4.3 Environment

These variables primarily relate to the child's family or the environments in which they lived. These included family income source (dichotomized into income from sources other than work or income from part- or full-time work); family composition (dichotomized into a 2 caregiver household or other household composition); whether the maltreatment was a form of physical punishment; whether a caregiver was one of the alleged perpetrators of maltreatment; functioning problems in any of the caregivers (alcohol abuse, drug abuse, ID, mental health issue, physical health problem, lack of social support, and intimate partner violence victimization); and whether the initial report came from a professional.

#### 2.4.4 Child protective services (CPS) and decisions

Variables pertaining to the CPS and decisions included: the child’s safety or development being compromised, number of prior CPS investigations, police involvement, service referrals, and the child’s placement throughout the investigation.

### 2.5 Analytic strategy

Descriptive statistics comparing autistic children and non-autistic children were calculated. Unadjusted binary logistic regressions were then conducted to assess effect size. Next, all independent variables that associated with a CPS-reported autism diagnosis that had a significance level of *p* < 0.25 were included in a multivariate binary logistic regression analysis, following the recommendation of Hosmer, Lemeshow, and Sturdivant (31), with autism status as the outcome variable. Evaluation of tolerance and VIF estimates did not reveal any indications of potential multicollinearity issues. Only the variables that remained significant at the *p* < 0.05 level were retained in the multivariate model. Subsequently, all the independent variables initially excluded from the multivariate model were reintroduced individually, and their significance at the conventional *p* < 0.05 threshold was assessed. If any of these variables proved to be significant, they were reintegrated into the model. To better interpret the strength of the odds ratios (OR) obtained, we have used Chen, Cohen, and Chen (32) suggestion of equivalence to Cohen's d effect sizes, and we applied an equivalent odds ratio (OR) based on a 4% outcome rate, considering that we had 4.0% with CPS reported autism diagnosis. Thus, ORs of 1.5, 3.0, and 5.0 were considered equivalent to Cohen's d effect sizes of 0.2 (small), 0.5 (medium), and 0.8 (large), respectively (32). These indicators allow a better estimate of the effect sizes observed and thus, a better identification of variables that distinguish maltreated cases of autistic and non-autistic children.

## 3 Results

Out of the 1,805 investigations where child maltreatment was substantiated, 73 cases (4.0%) involved children with a CPS-reported autism diagnosis. The results of the descriptive analyses, and the bivariate analyses are presented in Table 1. As can be seen in this table, all children, with or without autism, experienced at least one form of child maltreatment; the sum of these percentages is more than 100% because some children were identified as having experienced multiple forms of child maltreatment. Moreover, all autistic children experienced at least one issue related to child functioning, in contrast to 67.0% of the other non-autistic children (*χ*^2^ = 35.293, *p* < .000).

Results of the bivariate analyses (see Table 1) revealed that among the individual factors, CPS-reported autistic children were more likely to be boys. Regarding child maltreatment, autistic children experienced lower levels of psychological maltreatment. Significantly more problems were noted among autistic children: self-destructive behaviors, ADD/ADHD, attachment disorder, aggressive behaviors, physical disability and ID. Among the environmental factors, we found significantly higher levels of mental and physical health problems in one of the caregivers of the autistic children compared to the non-autistic children. Those caregivers were also significantly more likely to lack social support. Finally, regarding the CPS services and/or decisions, the safety or development were significantly more likely to be compromised.

The findings from the multivariate logistic regression analysis (see Table 2) provide insights into the factors that most prominently differentiate maltreated children with autism from those without, namely: being a boy; having ADD or ADHD, having an attachment disorder and ID in the child; and lower number of previous investigations. Two significant factors demonstrated substantial effect sizes in line with the statistical effect size standards established by Chen and his colleagues: being a boy (OR = 5.747), and ID (OR = 11.987). The other variables had small effect sizes (i.e., 1.4 ≤ OR < 3.0).

**Table 2:** Results of the multivariate analysis comparing maltreated autistic children to children without autism.

Variables	Multivariate analyses	
	B (SE)	OR [95% CI]
Individual		
Boys	1.749 (0.36)***	5.747 [2.835–11.652]
ADD/ADHD in the child	0.792 (0.28)**	2.207 [1.266–3.846]
Attachment disorder in the child	1.065 (0.28)***	2.899 [1.665–5.048]
ID in the child	2.484 (0.34)***	11.987 [6.177–23.261]
Service-related		
Number of past investigations	−0.326 (0.12)**	0.722 [0.569–0.916]

B, coefficient *b*; SE, standard error; OR, odd ratio; CI, confidence interval; x, mean; SD, standard deviation.

Final Model X2 (5) = 119.137. *p* < .000, Nagelkerke *R*^2^ = 0.231.

*p < .05; **p < .01; ***p < .001.

## 4 Discussion

The aim of this study was to examine prevalence of autism among substantiated cases of child maltreatment and to compare the characteristics of cases of children with a CPS-reported autism diagnosis compared to those without. Overall, 4% of children had CPS-reported autism in our sample of substantiated cases of child maltreatment aged 2–17. When compared to the prevalence of autism in the general population, our results suggest an overrepresentation of children with autism among those involved with child protection agencies for child maltreatment. This observation aligns with prior research that highlights the significance of child maltreatment as a concern for children with autism [see (25) for a scoping review]. These results differ from Sullivan & Knutson (33), but it should also be noted that their study had been conducted in 1994–1995, at a time where autism was less frequently diagnosed (34).

Among the sociodemographic characteristics, only being a boy remained significant in the multivariate analysis: maltreated boys, compared with maltreated girls, were more likely to be reported as autistic, mirroring overall gender differences in diagnoses for autism (close to 3:1 according to (35). All the referred autistic children also presented with at least one co-occurring condition. This finding is similar to the study of Hansen et al. (36), who found that 92% of Norwegian autistic children referred to child and adolescent mental health services had at least one additional comorbid disorder. In our study, autistic children had higher levels of self-destructive behaviors, ADD/ADHD, attachment disorder, aggressive behaviors, physical disability and ID compared to non-autistic children in the bivariate analyses. These mental health conditions are often co-occurring among autistic children (11) and adults (21). In the multivariate analyzes, only three of these functioning issues came out as significantly more frequent among autistic children compared to non-autistic children, namely, ADD/ADHD, attachment issues, and ID. Although they reflect distinct difficulties, we should note that autism and ADHD have shared genetic heritability and overlap in the impairments regarding social functioning and executive functioning (37, 38) which may explain these results. In another study of 3,331 Scottish children aged 5–6 years, although autism and symptoms of trauma- and stressor-related disorders (such as reactive attachment disorder and disinhibited social engagement disorder) were rare, there was a notable overlap between the symptoms associated with these disorders (39). While autistic children can show signs of secure attachment and prefer their mothers over strangers in the strange situation task, they are also engaged in less contact seeking and maintaining than other children (40, 41). Consequently, given these qualitative differences in early attachment markers, it is possible that these findings suggest how autistic children are more likely to be misdiagnosed with attachment problems. Lastly, due to the cross-sectional nature of the data collected, it remains unclear whether these functioning issues occurred prior to or subsequent to the occurrence of child maltreatment. Indeed, some researchers have argued that the consequences of early neglect and trauma can mimic autistic traits, or occasionally mischaracterized as “quasi-autism patterns” (i.e., patterns of behavior and affect characterized by attachment irregularities, deficits in social skills, perseverative behavior or detached and flat affect) (42). Results of a recent study, however, concluded that autism traits are independent of child maltreatment (25). In that context, it is crucial that practitioners be trained to better evaluate functioning problems among children who have experienced maltreatment.

Our results about children with a CPS-reported autism diagnosis presenting more often with ID than children without autism correspond with findings from the general population. For example, in Fisher et al., study (2019), 39% of autistic children also presented with an IQ below 70 (an IQ below 70 is one of the diagnosing criteria for ID). This co-occurrence of autism and ID may further increase the risk for maltreatment, as it may be more difficult for parents to take care of these children. Nevertheless, without having ID, many autistic children are considered non-verbal or minimally verbal, which may increase communication difficulties to disclose abuse.

Although the caregivers of autistic children reported higher levels of some problems than other caregivers (mental and physical health problems, and lack of social support), these factors did not remain significant in the multivariate analysis. These results may suggest that the factors that differentiate between autistic children and other children in child protective services may be stronger at the individual rather than at the family level. However, it is of note that regardless of having autism or not, all of the maltreated children and their families in this study in general faced substantial difficulties. Families of autistic children may also present with other difficulties not assessed in this study. For example, a high proportion of parents of autistic children have autistic traits, or even autism diagnoses (43). In light with Fisher's ecological model (2008), further studies should examine family functioning more generally to expand our knowledge of the families of autistic children.

At the bivariate level, autistic children appeared to experience lower levels of psychological maltreatment. This difference that was observed did not remain significant in the multivariate analysis. In fact, our study did not find any differences at the multivariate level across autism diagnoses with regard to the form of abuse. These findings are different from those of a previous study which showed physical abuse as being overrepresented among autistic children and underrepresented among children without a disability diagnosis (7). McDonnell et al. (9) has also found differences according to four different comparison groups: autism-only, autism and ID, ID-only and children with neither autism nor ID. Compared to the group with neither diagnosis, all groups were more likely to report physical neglect and physical abuse, and the autism-ID and the ID-only groups, all forms of abuse. Considering the importance of no longer studying autistic children as a homogeneous group (44), and taking into account that autistic children's intelligence may be underestimated and not easily assessed (45, 46), further studies including larger samples of different profiles of autistic children are needed to better understand variation in forms of child maltreatment in relation to autism, cognitive impairment, and other neurodevelopmental conditions.

In this current study, autistic children showed few differences from other children in terms of child protection decisions. Although the safety or development were significantly more likely to be compromised among autistic children at the bivariate level, this result was not significant at the multivariate level. In fact, our results indicate that a smaller percentage of families with autistic children were previously engaged with CPS (e.g., the number of past investigations was lower) compared to non-autistic children. These results might indicate that children with autism are less prone to encountering additional incidents or forms of maltreatment that would lead to their re-referral to CPS. This finding should be investigated further, especially given the functioning issues (e.g., ADHD, aggressive behaviors) among children with a CPS-reported autism diagnosis in this sample. Considering that these co-occurring factors in general increase the risk of maltreatment recurrence or chronicity, the current findings may suggest that autistic children are underserviced with regards to child protection. Nevertheless, our findings differ from those of previous studies in which children with disabilities were more likely to be re-referred to child protection or placement (24, 47). A potential limitation of our study was in how information was only available at the moment of the investigation. For example, placement following the investigation, which may occur more frequently compared to placement during investigation, was not evaluated. Further studies are thus needed to examine subsequent services received (or not) by autistic children, and on how to better answer to their needs. For instance, results of a recent meta-analysis suggest that placement may not be sufficient to decrease children's behavioral difficulties experienced by maltreated children (48). Given that the placement of autistic children may even be more challenging considering their unique needs for support, it would be important to know how this placement impacts their recovery.

### 4.1 Strengths and limitations

The current study had multiple strengths including a large sample size of child maltreatment cases with which to conduct multivariate analyses. Despite these advantages, the number of children with a CPS-reported autism diagnosis in this sample was still small, which may have limited our ability to detect differences in some of the lower prevalence variables, i.e., sexual abuse. The adoption of a cross-sectional design in this study has limited the extent to which the findings can be generalized. Although longitudinal studies are needed to better understand the direction of the relations between the variables studied, this research is one of the first to study substantiated cases of maltreated autistic children reported to CPS. As we solely considered substantiated cases, we could not examine child maltreatment not reported to CPS, which might differ from substantiated cases. Finally, the identification of autism relied on the CPS workers who compiled the record, and we also considered suspected diagnoses. Therefore, our study results are based on CPS-reported autism diagnosis; these were not formally established or assessed during the data collection. Moreover, accurate identification of autism is challenging (49). The symptoms of autism can vary widely in type and severity over time, and may be complicated by the presence of other neurodevelopmental disorders. This is further compounded by the high rates of comorbidity between autism and other conditions. Although our study was an important first step to better understand autistic children involved in CPS services, studies including formal proof of the autism diagnosis is the next step to confirm our results. These studies should also take into consideration other neurodevelopmental problems, using a holistic approach (12).

## 5 Conclusion

Overall this study aimed at better understanding maltreated autistic children in CPS. Results indicate a higher percentage of autistic children among these CPS cases than in the general population, suggesting a higher risk of child maltreatment. Moreover, our results indicate that what seems to distinguish autistic children more prominently from non-autistic children are characteristics specific to the autism condition rather than variables associated with services. For instance, autistic children with verified maltreatment presented with more co-occurring conditions than identified non-autistic children: more ID, attachment problems, and ADD/ADHD, which suggest that they may have increased service needs when compared with service users more broadly. However, autistic children were not more frequently referred to services compared to other children. We may wonder if it is because specific services (and therapist expertise) for autistic children are lacking, as has been found in a systematic review on barriers to seeking and accessing help for mental health or behavioral problems in autistic individuals (50). As the heightened likelihood of child maltreatment with disability arises from a complex interplay of social, cultural, psychological, economic, and various other contributing factors (27), further studies are needed to better understand systemic factors that may play a pivotal role in the child maltreatment of autistic children and services they receive (or not). Moreover, our findings suggest that our society needs to develop CPS services inclusive of the specific needs of autistic children, in order to offer them with a more effective response, assessment and intervention strategies (51).

## Data Availability

The raw data supporting the conclusions of this article will be made available by the authors, without undue reservation.

## Footnotes

^1^Paquette G, Bolduc M-L, De La Sablonnière-Griffin M, Argumedes M, Dion J. *Understanding Maltreatment among Autistic Children: Insights for Practitioners and Future Research Directions, United States* (submitted).

## References

[1] Widom CS. Longterm consequences of child maltreatment. In: Korbin J, Krugman R, editors. Handbook of Child Maltreatment. Child Maltreatment. Vol 2. Dordrecht: Springer (2014). 10.1007/978-94-007-7208-3_12

[2] World Health Organization. Child Maltreatment. Geneva: World Health Organization (2016). Available online at: http://www.who.int/mediacentre/factsheets/fs150/en/

[3] Amborski AM Bussières E-L Vaillancourt-Morel M-P Joyal CC. Sexual violence against persons with disabilities: a meta-analysis. Trauma Violence Abuse. (2022) 23(4):1330–43. 10.1177/1524838021995975 33657931 PMC9425723

[4] Jones L Bellis MA Wood S Hughes K McCoy E Eckley L Prevalence and risk of violence against children with disabilities: a systematic review and meta-analysis of observational studies. Lancet (London, England). (2012) 380(9845):899–907. 10.1016/S0140-6736(12)60692-8 22795511

[5] Zeidan J Fombonne E Scorah J Ibrahim A Durkin MS Saxena S Global prevalence of autism: a systematic review update. Autism Res. (2022) 15(5):778–90. 10.1002/aur.2696 35238171 PMC9310578

[6] Fisher MH Epstein RA Urbano RC Vehorn A Cull MJ Warren Z. A population-based examination of maltreatment referrals and substantiation for children with autism spectrum disorder. Autism. (2019) 23(5):1335–40. 10.1177/136236131881399 30523699 PMC6555697

[7] Hall-Lande J Hewitt A Mishra S Piescher K LaLiberte T. Involvement of children with autism spectrum disorder (ASD) in the child protection system. Focus Autism Dev Dis. (2015) 30(4):237–48. 10.1177/1088357614539834

[8] Dinkler L Lundström S Gajwani R Lichtenstein P Gillberg C Minnis H. Maltreatment-associated neurodevelopmental disorders: a co-twin control analysis. J Child Psychol Psychiatry. (2017) 58(6):691–701. 10.1111/jcpp.12682 28094432

[9] McDonnell CG Boan AD Bradley CC Seay KD Charles JM Carpenter LA. Child maltreatment in autism spectrum disorder and intellectual disability: results from a population-based sample. J Child Psychol Psychiatry. (2019) 60(5):576–84. 10.1111/jcpp.12993 30368827 PMC6458088

[10] Fisher MH Hodapp RM Dykens EM. Child abuse among children with disabilities: what we know and what we need to know. Int Rev Res Ment Retard. (2008) 35:251–89. 10.1016/s0074-7750(07)35007-6

[11] Hollander E Hagerman R Ferretti C. Textbook of Autism Spectrum Disorders. Washington, DC: American Psychiatric Association Publishing (2022).

[12] Gillberg C. The ESSENCE in child psychiatry: early symptomatic syndromes eliciting neurodevelopmental clinical examinations. Res Dev Disabil. (2010) 31(6):1543–51. 10.1016/j.ridd.2010.06.002 20634041

[13] Hayes SA Watson SL. The impact of parenting stress: a meta-analysis of studies comparing the experience of parenting stress in parents of children with and without autism spectrum disorder. J Autism Dev Disord. (2013) 43(3):629–42. 10.1007/s10803-012-1604-y 22790429

[14] Kerns CM Newschaffer CJ Berkowitz S Lee BK. Brief report: examining the association of autism and adverse childhood experiences in the national survey of children’s health: the important role of income and co-occurring mental health conditions. J Autism Dev Disord. (2017) 47:2275–81. 10.1007/s10803-017-3111-7 28378271 PMC5523127

[15] Brenner J Pan Z Mazefsky C Smith KA Gabriels R, for the Autism and Developmental Disorders Inpatient Research Collaborative (ADDIRC). Behavioral symptoms of reported abuse in children and adolescents with autism spectrum disorder in inpatient settings. J Autism Dev Disord. (2018) 48(11):3727–35. 10.1007/s10803-017-3183-4 28593599

[16] Ahmed FE. Extents of abuse and behavioural disorders in autistic children who were abused and who were not abused. Cypriot J Educ Sci. (2021) 16(1):114–28. 10.18844/cjes.v16i1.5513

[17] Mandell DS Walrath CM Manteuffel B. Characteristics of children with autistic spectrum disorders served in comprehensive community-based mental health settings. J Autism Dev Dis. (2005) 35(3):313–21. 10.1007/s10803-005-3296-z 16119472

[18] DeFilippis M. Depression in children and adolescents with autism spectrum disorder. Children. (2018) 5(9):112. 10.3390/children5090112 30134542 PMC6162511

[19] Houghton R Liu C Bolognani F. Psychiatric comorbidities and psychotropic medication use in autism: a matched cohort study with ADHD and general population comparator groups in the United Kingdom. Autism Res. (2018) 11(12):1690–700. 10.1002/aur.2040 30380202

[20] Leyfer OT Folstein SE Bacalman S Davis NO Dinh E Morgan J Comorbid psychiatric disorders in children with autism: interview development and rates of disorders. J Autism Dev Disord. (2006) 36(7):849–61. 10.1007/s10803-006-0123-0 16845581

[21] Lugo-Marin J Magan-Maganto M Rivero-Santana A Cuellar-Pompa L Alviani M Jenaro-Rio C Prevalence of psychiatric disorders in adults with autism spectrum disorder: a systematic review and meta-analysis. Res Autism Spectr Disord. (2019) 59:22–33. 10.1016/j.rasd.2018.12.004

[22] Franklin A Toft A Hernon J Greenaway J Goff S. UK social Work Practice in Safeguarding Disabled Children and Young People. A Qualitative Systematic Review. London: What Works for Children’s Social Care (2022).

[23] Hoover DW. The effects of psychological trauma on children with autism spectrum disorders: a research review. Rev J Autism Dev Dis. (2015) 2(3):287–99. 10.1007/s40489-015-0052-y

[24] Cidav Z Xie M Mandell DS. Foster care involvement among medicaid-enrolled children with autism. J Autism Dev Disord. (2018) 48(1):176–83. 10.1007/s10803-017-3311-1 28929296

[25] Mayes SD Breaux RP Calhoun SL Whitmore K. History of maltreatment is not associated with symptom profiles of children with autism. J Dev Phys Disabil. (2019) 31(5):623–33. 10.1007/s10882-019-09661-9

[26] Pfeffer RD. Childhood victimization in a national sample of youth with autism spectrum disorders. J Policy Pract Intellect Disabil. (2016) 13(4):311–9. 10.1111/jppi.12203

[27] Flynn S. Theorizing disability in child protection: applying critical disability studies to the elevated risk of abuse for disabled children. Disabil Soc. (2020) 35(6):949–71. 10.1080/09687599.2019.1669433

[28] Hélie S Collin-Vézina D Turcotte D Trocmé N Girouard N. Étude D'incidence Québécoise sur les Situations évaluées en Protection de la Jeunesse en 2014:(ÉIQ-2014). Quebec: Centre jeunesse de Montréal-Institut universitaire (2017).

[29] Stone WL Lee EB Ashford L Brissie J Hepburn SL Coonrod EE Can autism be diagnosed accurately in children under 3 years? J Child Psychol Psychiatry. (1999) 40:219–26. 10.1111/1469-7610.00435 10188704

[30] De La Sablonnière-Griffin M Paquette G Hélie S Dion J. Child maltreatment investigations and substantiations in child protection services: factors distinguishing children with intellectual disabilities. Disabil Health J. (2021) 14(4):101128. 10.1016/j.dhjo.2021.101128 34144896

[31] Hosmer DW Jr Lemeshow S Sturdivant RX. Applied Logistic Regression. 3rd ed. Hoboken, NJ: John Wiley & Sons, Inc (2013). 10.1002/9781118548387

[32] Chen H Cohen P Chen S. How big is a big odds ratio? Interpreting the magnitudes of odds ratios in epidemiological studies. Commun Stat Simulation Comp. (2010) 39(4):860–4. 10.1080/03610911003650383

[33] Sullivan PM Knutson JF. Maltreatment and disabilities: a population-based epidemiological study. Child Abuse Negl. (2000) 24(10):1257–73. 10.1016/s0145-2134(00)00190-3 11075694

[34] Hansen SN Schendel DE Parner ET. Explaining the increase in the prevalence of autism spectrum disorders: the proportion attributable to changes in reporting practices. JAMA Pediatr. (2015) 169(1):56–62. 10.1001/jamapediatrics.2014.1893 25365033

[35] Loomes R Hull L Mandy WPL. What is the male-to-female ratio in autism spectrum disorder? A systematic review and meta-analysis. J Am Acad Child Adolesc Psychiatry. (2017) 56(6):466–74. 10.1016/j.jaac.2017.03.013 28545751

[36] Hansen BH Oerbeck B Skirbekk B Petrovski BÉ Kristensen H. Neurodevelopmental disorders: prevalence and comorbidity in children referred to mental health services. Nord J Psychiatry. (2018) 72(4):285–91. 10.1080/08039488.2018.1444087 29488416

[37] Antshel KM Russo N. Autism spectrum disorders and ADHD: overlapping phenomenology, diagnostic issues, and treatment considerations. Curr Psychiatry Rep. (2019) 21(5):1–11. 10.1007/s11920-019-1020-5 30903299

[38] Leitner Y. The co-occurrence of autism and attention deficit hyperactivity disorder in children–what do we know? Front Hum Neurosci. (2014) 8(268):1–8. 10.3389/fnhum.2014.00268/full 24808851 PMC4010758

[39] Minnis H Messow CM McConnachie A Bradshaw P Briggs A Wilson P Autism and attachment disorder symptoms in the general population: prevalence, overlap, and burden. Dev Child Welfare. (2020) 2(1):37–51. 10.1177/2516103220902778 PMC13021084 41907538

[40] Cossette-Côté F Bussières EL Dubois-Comtois K. The association between maternal sensitivity/availability and attachment in children with autism spectrum disorder: a systematic review and meta-analysis. Curr Psychol. (2021) 41:1–13. 10.1007/s12144-021-02227-z PMC8412397 34493913

[41] Rutgers AH Bakermans-Kranenburg MJ Ijzendoorn MH Berckelaer-Onnes IA. Autism and attachment: a meta-analytic review. J Child Psychol Psychiatry. (2004) 45(6):1123–34. 10.1111/j.1469-7610.2004.t01-1-00305.x 15257669

[42] Hoover DW. Trauma in children with neurodevelopmental disorders: autism, intellectual disability, and attention-deficit/hyperactivity disorder. In: Spalletta G Janiri D Piras F Sani G, editors. Childhood Trauma in Mental Disorders. Cham: Springer International Publishing (2020). p. 367–83. 10.1007/978-3-030-49414-8_17

[43] Constantino JN Todd RD. Intergenerational transmission of subthreshold autistic traits in the general population. Biol Psychiatry. (2005) 57(6):655–60. 10.1016/j.biopsych.2004.12.014 15780853

[44] Mottron L. A radical change in our autism research strategy is needed: back to prototypes. Autism Res. (2021) 14(10):2213–20. 10.1002/aur.2494 34077611

[45] Dawson M Soulières I Ann Gernsbacher M Mottron L. The level and nature of autistic intelligence. Psychol Sci. (2007) 18(8):657–62. 10.1111/j.1467-9280.2007.01954.x 17680932 PMC4287210

[46] Soulières I Dawson M Gernsbacher MA Mottron L. The level and nature of autistic intelligence II: what about asperger syndrome? PLoS One. (2011) 6(9):e25372. 10.1371/journal.pone.0025372 21991394 PMC3182210

[47] Connell CM Bergeron N Katz KH Saunders L Tebes JK. Re-referral to child protective services: the influence of child, family, and case characteristics on risk status. Child Abuse Negl. (2007) 31(5):573–88. 10.1016/j.chiabu.2006.12.004 17537504

[48] Dubois-Comtois K Bussières EL Cyr C St-Onge J Baudry C Milot T Are children and adolescents in foster care at greater risk of mental health problems than their counterparts? A meta-analysis. Child Youth Serv Rev. (2021) 127:106100. 10.1016/j.childyouth.2021.106100

[49] Hus Y Segal O. Challenges surrounding the diagnosis of autism in children. Neuropsychiatr Dis Treat. (2021) 17:3509–29. 10.2147/NDT.S282569 34898983 PMC8654688

[50] Adams D Young K. A systematic review of the perceived barriers and facilitators to accessing psychological treatment for mental health problems in individuals on the autism spectrum. Rev J Autism Dev Dis. (2021) 8:1–18. 10.1007/s40489-020-00226-7

[51] Kendall-Tackett K Lyon T Taliaferro G Little L. Why child maltreatment researchers should include children’s disability status in their maltreatment studies. Child Abuse Negl. (2005) 29(2):147–51. 10.1016/j.chiabu.2004.09.002 15734180

